# 

**Published:** 1980

**Authors:** 


JULY/OCTOBER 1980

VOLUME 95 (iii/iv)

NUMBER 355/356

Published Quarterly

Annual Subscription £6 Post Free

### BRISTOL MEDICO-CHIRURGICAL JOURNAL

Contents

3 **Editorial**

Established 1883

### BRISTOL MEDICO-CHIRURGICAL JOURNAL

Incorporating the Medical Journal of the South West

### Editorial Committee

EDITOR
*M. G. Wilson, Esq.*
*Litfield House, Clifton, Bristol, BS8 3LS*

ASSISTANT EDITOR
*Dr. G. R. Tribe*
*Southmead Hospital, Bristol, BS10 5NB*

MEMBERS
*Dr. Rhys Davies*
*Dr. M. B. Lennard*
*Dr. D. W. Wright*
*The Hon. Treasurer of the Society*
*The Hon. Secretary of the Society*

SECRETARY
*Mr. B. P. Jones, The Library, Medical School, University Walk, Bristol, BS8 1TD*

### Bristol Medico-Chirurgical Society

PRESIDENT 1980/81
*Dr. N. J. Brown*

PRESIDENT ELECT
*Dr. R. P. Warin*

HONORARY SECRETARY
*Dr. H. M. Norman*
*Hillside, Vicarage Lane, Barrow Gurney*

HONORARY TREASURER
*Dr. J. Penry*
*Southmead Hospital, Bristol, BS10 5NB*

The Society was founded in 1874. In 1883 publication of the Journal began and has continued quarterly ever since then. During the years 1949 to 1962 it appeared under the title of 'The Medical Journal of the South West'.

### Notice to Contributors

*The Journal is especially concerned to provide a place for the recording of medical thought, interests, and practice in and around Bristol. It therefore welcomes articles that, as well as being of immediate interest to members, will document the local and contemporary medical scene for our successors.*

*Original articles are invited provided that they have not been published elsewhere. Illustrations should be supplied ready for reproduction. Line drawings should be in Indian ink on firm white paper or board. The author will be informed if it is found necessary to ask him to pay for the printing of photographs, etc.*

*The following contributions will be especially welcome: notes of forthcoming events, meetings held, degrees conferred, staff appointments, honours and public appointments and other local news.*

*Advice on preparation can be obtained from the Editor.*

Printed by the University of Bristol Printing Unit

### University of Bristol Faculty of Medicine

**THE DEGREE OF MASTER OF SCIENCE IN MEDICAL SCIENCES**
**October 1980**
**Dissertations Approved**

Ali Ghassemi ISFAHANI
Mahmood Muhammed KHALAF
Mohammed Hussain Abid Ali RAHEEM

**THE DEGREE OF DOCTOR OF PHILOSOPHY**
**June 1980**
**Dissertations Approved**

Patricia Ann EVANS
David Clement John MAIDMENT

**THE DEGREE OF DOCTOR OF PHILOSOPHY**
**October 1980**
**Dissertation Approved**

Peter Philip ROBINSON

**THE DEGREE OF DOCTOR OF PHILOSOPHY**
**October 1980**
**Published Work Approved**

Hamish Roy DENNY

**FINAL EXAMINATION FOR THE DEGREES OF M.B., Ch.B.**
**June 1980**

**WITH HONOURS**
Robin Jeffrey BALL
with Distinction in Mental Health
Sarah Renata DERBYSHIRE
with Distinction in Mental Health
Andrew FOULKES
with Distinction in Surgery
Jacqueline Patricia GUMB
with Distinction in Obstetrics & Gynaecology
Judith Elizabeth JONES
with Distinctions in Pathology and in Community Health
Anne Elizabeth MEDILL
with Distinction in Pathology

**PASS**
Geoffrey Phillip ADAMS
Ian Thomas Kassam ADATIA
Amanda Jane ADDISON
with Distinction in Community Health
Susan Rowena Felicity ANSLOW
Gillian Frances BAILEY
Peter John BANYARD
Charles Simon BARKER
John Christopher BASS
Susan Hilary BEDFORD
Sheila Ann BODDY
Karen Joy BOUCHER
Lyn BRADLEY
John Philip BRITTON
Kathryn Jane BROOKS
David Ian BRUCE
Elizabeth BURD
Giles Victor CAMPION
with Distinction in Child Health
Jonathan CHAMBERS
Brigitte Florise CHEUNG FOON CHEONG
Simon Richard CHILD
Thomas Henry CLUTTON-BROCK
Anthony John COLLINS
Marilyn Anne COOK
Timothy Ralph Carless DAVIS
Jacqueline Karen DAY
Romain DE COCK
Christine Elizabeth DUNCUMB
Nuala Mary DUNNE
Deirdre Ann Elizabeth DURRANT
with Distinction in Obstetrics & Gynaecology
Julia Anne EDGE
Keith Leonard FARREL
Nicola Jane FOTHERGILL
Alison Mary FOX
Christopher Richard Thomas FRANCIS
Oliver Hugh Basil FRANKS
Adrian Peter GALLIMORE
Katharine Jean GORDON
Timothy John GOULD
Margaret GREGORY
Deborah Susan HACON
Ruth Jasmine HARKER
Graham Alan John HARRISON
Stephen John HAWES
Kim Pauline HEARN
Sarah HEARSE
Paul David HOLDER
Bronwen Ruth HUGHES
Madho JINGREE
Alison Louise JONES

Julian Eamon KABALA
Trevor John KEELING
Caroline Jane KENNEDY-COOKE
Stephen Randolph KISELY
Janet Anne KNOWLES
Paul KOROS
Elisabeth KUTT
Charles Vincent LANSLEY
Stephen LEADBEATTER
Helen LUNT
John MAHONY
Clare Jennifer MATTINGLY
Deirdre Susan MERCER
Nigel Stuart George MERCER
Timothy John Fitzmaurice MITCHELL
Wendy Barbara MITCHELL
Patricia Mary MUNSON
Lina NASHEF
Samer Abdul Malik NASHEF
Christopher Choon Ee NEOH
Peter William PAIRAUDEAU
Timothy John PARKE
with Distinction in Mental Health
Gillian Mary PATTERSON
Gareth Huw PHILLIPS
Sheila Christine PIETERSEN
Stephen Howard Christopher PILL
Mary Bernadette PILLAI
with Distinction in Child Health
Martin John QUINN
Christine Ann RILEY
Gerard William ROBERTS
Jane Elisabeth ROBERTS
Susan SHEPHERD
with Distinction in Child Health
Michael Joseph William SLOT
Rosalyn May STANBURY
Andrew STORER
Mark Anthony STOTT
David Graham SWANN
David Julian TAWN
Ming Keng TEOH
John Lindsay TWEEDALE
Timothy John UNDERHILL
Mary Josephine VALENTINE
Stephen John VALENTINE
William Munro WEIGHTMAN
Alison Jean WICKERT
Adrian John WILLIAMS
with Distinction in Medicine
Andrew James Kevin WILLIAMS
Lynette WILLIAMS
John Michael Steele WILLIAMSON
Janet Diane WILSON
with Distinction in Mental Health
David Bruce YORSTON

